# Goodall's Elastic or Spring Plates

**Published:** 1869-05

**Authors:** 


*Goodall's Elastic or Spring Plates.*—In the Feb. number of the *Journal* an article was published from Dr. Goodall explaining his method of constructing these elastic or spring plates, and his claims for the patent.

Some practitioners, however, are disposed to doubt not only the utility of these plates indiscriminately inserted, but also the validity of the claim for their invention. The question has also been asked as to how they differ from those formerly in use constructed in the same manner. As proof that this method of inserting plates has been in use for many years, reference is made, in one of the articles published, to a paragraph in the first edition of "Richardson's Mechanical Dentistry, 1860, which reads as follows : "*Plastic or Stay Clasps.*—This form of clasp, instead of embracing the tooth, is designed to steady or fix the substitute in place by simply resting against one side of the tooth to which it is applied. They should be so connected to the plate that when pressed over the enlarged portion of the crowns of the teeth, they will spring readily into place and adapt themselves closely to the more contracted parts near the gum. In cases where there is no adequate opposing force to that exerted by the clasp, care should be taken that no more pressure is produced than is necessary to keep the substitute in place, as, without this precaution outward displacement of the teeth is liable to occur, and the appliance losing its bearing upon the teeth soon becomes loosened and insecure in the mouth. The result alluded to should be particularly guarded against in the case of young subjects whose teeth are easily moved by the application of very slight forces."

